# Heat transfer analysis of generalized second-grade fluid with exponential heating and thermal heat flux

**DOI:** 10.1038/s41598-023-44007-8

**Published:** 2023-10-07

**Authors:** Saeed Ullah Jan, Sami Ul Haq, Naseeb Ullah, Wasi Ullah, Sehra, Ilyas Khan

**Affiliations:** 1 Department of Mathematics, Islamia College Peshawar, Peshawar, 25000 Khyber Pakhtunkhwa Pakistan; 2 Shaheed Benazir Bhutto Women University Peshawar, Peshawar, 25000 Khyber Pakhtunkhwa Pakistan; 3 Department of Mathematics, College of Science Al-Zulfi, Majmaah University, 11952 Al-Majmaah, Saudi Arabia

**Keywords:** Mathematics and computing, Physics

## Abstract

The aim of present work is to apply the Caputo–Fabrizio fractional derivative in the constitutive equations of heat transfer. Natural convection flow of an unsteady second grade fluid over a vertical plate with exponential heating is discussed. The generalized Fourier law is substituted in temperature profile. A portion of the dimensionless factors are utilized to make the governing equations into dimensionless structures. The solutions for temperature and velocity profiles of Caputo–Fabrizio model are acquired through the Laplace transform method. These solutions are greatly affected through the variation of different dimensionless variables like Prandtl number, Grashof number, and second-grade fluid parameter. Finally, the influence of embedded parameters is shown by plotting graphs through Mathcad. From the graphical results it is concluded that, the temperature of the fluid decreases with the increasing values of the Prandtl number and Second grade fluid parameter and increases with the passage of time. The velocity of the fluid increases with increasing values of the Grashof number, second grade parameter and time while decreases with increasing values of fractional parameter and Prandtl number.

## Introduction

The study of non-Newtonian fluids in fluid mechanics is more useful than Newtonian fluids because of the intricate interaction between stress and strain in non-Newtonian fluids and its industrial and engineering applications. The extraction of energy from geothermal regions, the flow of oil through porous rocks, the filtering of particles from fluids, and medication penetration through human skin are all examples of non-Newtonian fluid applications. Second-grade fluids are non-Newtonian fluids that have up to a second derivative in the strain tensor relation, whereas Newtonian fluids have just a first derivative. Many researchers and engineers were interested in the flow of second-grade fluid. Nehad and Khan^1^ investigated the second-grade fluid passing on vertical plate utilizing Caputo–Fabrizio (CF) fractional derivative. Samiulhaq et al.^2^ studied the fractional second-grade fluid of MHD flow considering modified Darcy’s law and exponential heating. The LT is utilized for the solution of CF model. The influence of fractional order derivative having non-singular kernel on second-grade fluid was analyzed by Fetecau et al.^3^. The impact of second-grade fluid in vertical and circular cylinder was considered by Refs.^4,5^. The exact solution is acquired via Laplace transform (LT). References^6,7^ investigated of fractional immiscible multi-layer flow of second-grade fluid in rectangular channel. The remedy is acquired via finite Fourier sine and LT. Siddique et al.^8^ studied the heat transfer analysis of fractional second-grade fluid with Newtonian heating. CF and ABC fractional operators are introduced in the fluid model. Elnaqeeb et al.^9^ analyzed the natural convection flow of second-grade nanofluid using Prabhakar fractional operator for the solution. Abro^10^ investigated the second-grade MHD fluid with permeable medium utilizing CF fractional operator. References^11,12^ studied the fractional (Caputo, CF and ABC) rotational second-grade fluid with thermal stratification. The remedy is got via Laplace transform. Importance of magnetic field of second-grade fluid on the dynamical analysis was given by Riaz et al.^13^. The CF and ABC fractionalized model is solving through LT method. Sene^14^ investigated the generalized second-grade fluid using Caputo–Liouvile fractional operator. Nisa et al.^15^ studied the second-grade fluid with damped thermal flux and radiation among vertical channel. Generalized Fourier law has been utilized in energy equation. The solution is acquired from the most powerful method called Laplace transform. Haq et al.^16^ analyzed the fractional second-grade fluid with ramped wall heating and slippage effects on heat and mass transfer model. The CF time derivative is utilized in temperature profile. The solution is acquired via Laplace transform.

The above literature motivates us to work on natural convection flow of fractional second grade fluid with generalized Fourier’s law. The main novelty of this article is the analysis of heat transfer through natural convection under the effects of exponential heating with generalized Fourier thermal flux. The solution for velocity and temperature field is acquired via Laplace transform technique. The effects of different embedded parameters were finally visually and conceptually demonstrated.

## Problem’s formation

**Figure 1:**
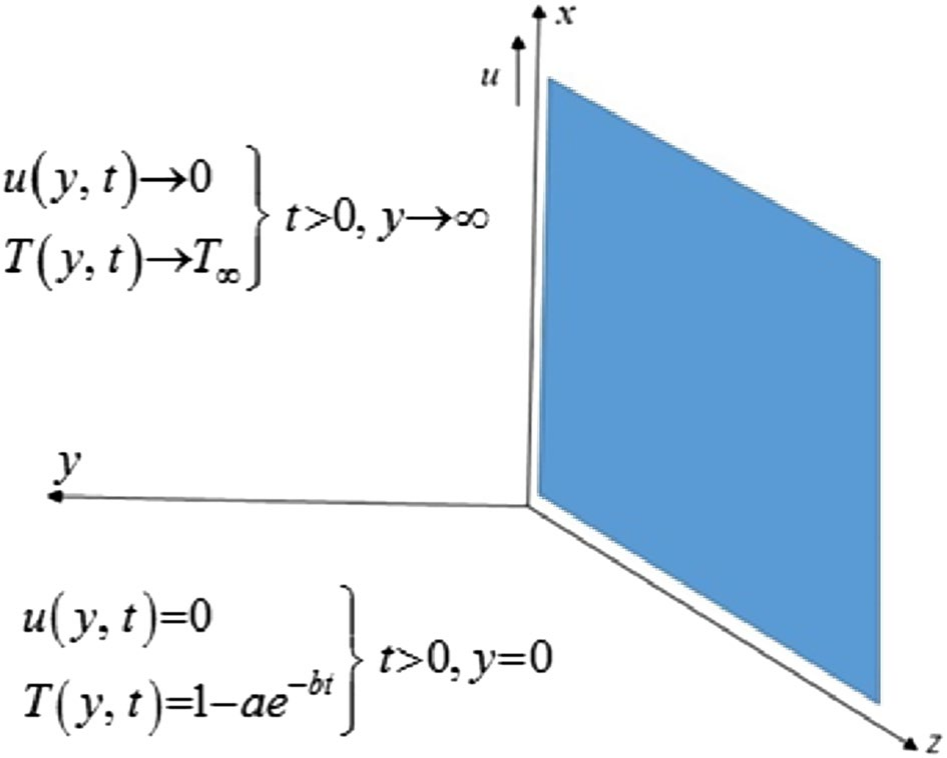
Physical model of the problem.

Consider the second grade fluid of unsteady flow closer to an infinite upright plate with exponential heating the straight upward direction of the plate. Consider as x-axis direction and y-axis is put up with perpendicular to the flat established by the plate when t = 0 both the fluid and the plate are remainder to the constant temperature T infinity. After some time, the plate begins to move at the specified speed. The governing equation is provided by, according to the standard Boussinesq’s approximation^17^. Fluid flow problem is represented geometrically by Fig. 1.

1 $$ \frac{\partial u}{{\partial t}} = \left( {\upsilon + \frac{\lambda }{\rho }\frac{\partial }{\partial t}} \right)\frac{{\partial^{2} u}}{{\partial y^{2} }} + g\beta \left( {T - T_{\infty } } \right), $$

2 $$ \rho c_{p} \frac{\partial T}{{\partial t}} = - \frac{\partial q}{{\partial y}}, $$

where $\upsilon ,\lambda ,\rho ,k,c_{p} ,\beta ,g$ are respectively kinematic viscosity, second-grade parameter, density, thermal conductivity, specific heat, thermal expansion and gravitational acceleration.

Following are the IC’s and BC’s;

3 $$ \begin{array}{*{20}l}    {u(y,t) = 0,{\text{   }}T(y,t) = T_{\infty } \,\,\,at\,\,t = 0} \hfill  \\    {u(y,t) = 0\,\,\,at\,\,y = 0,{\text{    }}T(y,t) = 1 - ae^{{ - bt}} \,\,at\,\,y = 0} \hfill  \\    {u \to 0,{\text{   }}T \to T_{\infty } ,{\text{ }}y \to \infty } \hfill  \\   \end{array}.  $$

Substituting the bellow dimensionless factors,

4 $$ \begin{array}{*{20}l} u^{*} = \frac{u}{U},{\text{ t}}^{*} = \frac{{U^{2} }}{\upsilon }t,{\text{ y}}^{*} = \frac{U}{\upsilon }y \hfill \\ \alpha_{1} = \frac{{U^{2} }}{\upsilon }\lambda , \, \theta = \frac{{\left( {T - T_{\infty } } \right)}}{{T_{\infty } }},{\text{ Pr}} = \frac{{\mu c_{p} }}{k} \hfill \\ Gr = \frac{{U^{3} g\beta T_{\infty } }}{{\upsilon^{2} }}. \hfill \\ \end{array} $$

Introducing the above unit less factor in (1)–(3) and drop the star, we acquired

5 $$ \frac{{\partial u\left( {y,t} \right)}}{\partial t} = \left( {1 + \alpha_{1} \frac{\partial }{\partial t}} \right)\frac{{\partial^{2} u\left( {y,t} \right)}}{{\partial y^{2} }} + Gr\theta \left( {y,t} \right). $$

The fractional generalization of Fourier law is given by Henry et al.^18^ and Hristov^19,20^, which addresses the thermal heat flux.

6 $$ q(y,t) = - kD_{t}^{\gamma } \left[ {\nabla T(y,t)} \right]. $$

Caputo–Fabrizio fractional derivative and its Laplace transform is defined as^21^

7 $$ \left. \begin{array}{*{20}l} {}^{CF}D_{{\overline{\tau }}}^{{\tilde{\gamma }}} \tilde{o}\left( {\overline{\tilde{Y}},\overline{\tau }} \right) = \frac{1}{{\left( {\dot{v} - \tilde{\gamma }} \right)}}\int\limits_{0}^{{\overline{\tau }}} {e^{{\left( { - \frac{{\tilde{\gamma }\left( {\tilde{\gamma } - \overline{\tau }} \right)}}{{1 - \tilde{\gamma }}}} \right)}} \partial_{{\tilde{s}}} } \tilde{o}\left( {\overline{\tilde{Y}},\tilde{s}} \right)d\tilde{s}, \hfill \\ L\left\{ {{}^{CF}D_{{\overline{\tau }}}^{{\tilde{\gamma }}} \tilde{o}\left( {\overline{\tilde{Y}},\overline{\tau }} \right)} \right\} = \overline{\tilde{o}}\left( {\overline{\tilde{Y}},\overset{\lower0.5em\hbox{$\smash{\scriptscriptstyle\smile}$}}{r} } \right) = \frac{{\overset{\lower0.5em\hbox{$\smash{\scriptscriptstyle\smile}$}}{r} L\left\{ {\tilde{o}\left( {\overline{\tilde{Y}},\overline{\tau }} \right)} \right\} - \tilde{o}\left( {\overline{\tilde{Y}},0} \right)}}{{\left( {1 - \tilde{\gamma }} \right)\overset{\lower0.5em\hbox{$\smash{\scriptscriptstyle\smile}$}}{r} + \tilde{\gamma }}} \hfill \\ \end{array} \right\}. $$

Using the above CF definition in Eq. (6) in $D_{t}^{\gamma }$ we obtain the following form,$\gamma$ is fractional parameter,

8 $$ \Pr \frac{\partial \theta (y,t)}{{\partial t}} = D_{t}^{\gamma } \frac{{\partial^{2} \theta (y,t)}}{{\partial y^{2} }}. $$

The dimensionless initial and boundary condition are follow;

9 $$  \begin{array}{*{20}l}    {u\left( {y,t} \right)\,\,\, = 0,{\text{    }}\theta \left( {y,t} \right)\,\,\, = 0\,\,\,\,\,at\,\,t\,\, = \,\,0} \hfill  \\    {u\left( {y,t} \right)\,\,\, = 0,{\text{    }}\theta \left( {y,t} \right)\,\,\, = 1 - a \cdot e^{{ - bt}} \,\,\,\,at\,\,y\,\, = \,\,0} \hfill  \\    {u \to 0,{\text{    }}\theta  \to 0,{\text{  }}y \to \infty } \hfill  \\   \end{array}.  $$

## Solution of the problem

### Temperature profile

Put Eq. (7) in Eq. (8) and taking Laplace transform to Eq. (8) and also to associated IC and BC, we acquired

10 $$ \frac{{d^{2} \overline{\theta }\left( {y,q} \right)}}{{dy^{2} }} - \frac{{\Pr \left( {q + \gamma a_{1} } \right)}}{{a_{1} }}\overline{\theta }\left( {y,q} \right) = 0, $$

where $$a_{1} = \frac{1}{1 - \gamma }.$$

The transform IC and BC are following,

11 $$ L\left\{ {1 - ae^{ - bt} } \right\} = \frac{1}{q} - \frac{a}{b + q}. $$

After solving Eq. (10) and using Eq. (11), we acquired

12 $$ \overline{\theta }\left( {y,q} \right) = \left[ {\frac{1}{q} - \frac{a}{b + q}} \right] \cdot e^{{ - y\sqrt {\frac{{\Pr (q + \gamma a_{1} )}}{{a_{1} }}} }} . $$

Equation (12) inverse Laplace transformation is as under:

13 $$ \theta = \Phi (y\,,t,a_{1} ,\gamma ,\,\,\Pr ) - a\Theta \left( {\,y,t,a_{1} ,\gamma ,\Pr } \right), $$

where

14 $$ \Phi \left( {y,t,a_{1} ,\gamma ,\Pr } \right) = \frac{1}{2}\left[ {e^{{ - y\left( {\sqrt {\Pr \gamma } } \right)}} erfc\left\{ {\frac{{y\sqrt {\Pr } }}{{2\sqrt {a_{1} t} }} - \sqrt {\gamma a_{1} t} } \right\} + e^{{y\left( {\sqrt {\Pr \gamma } } \right)}} erfc\left\{ {\frac{{y\sqrt {\Pr } }}{{2\sqrt {a_{1} t} }} + \sqrt {\gamma a_{1} t} } \right\}} \right], $$

and

15 $$ \Theta \left( {y,t,a_{1} ,\gamma ,\Pr } \right) = \frac{{e^{ - bt} }}{2}\left[ {e^{{ - y\left( {\sqrt {\frac{{\Pr \left( {\gamma a_{1} - b} \right)}}{{a_{1} }}} } \right)}} erfc\left\{ {\frac{{y\sqrt {\Pr } }}{{2\sqrt {ta_{1} } }} - \sqrt {\left( {\gamma a_{1} - b} \right)t} } \right\} + e^{{y\left( {\sqrt {\frac{{\Pr \left( {\gamma a_{1} - b} \right)}}{{a_{1} }}} } \right)}} erfc\left\{ {\frac{{y\sqrt {\Pr } }}{{2\sqrt {ta_{1} } }} + \sqrt {\left( {\gamma a_{1} - b} \right)t} } \right\}} \right]. $$

### Velocity profile

Taking LT on Eq. (5) and their associated IC and BC, and substitute Eq. (13) we acquired the following ODE,

16 $$ \frac{{d^{2} \overline{u}\left( {y,q} \right)}}{{dy^{2} }} - \left( {\frac{q}{{1 + \alpha_{1} q}}} \right)\overline{u}\left( {y,q} \right) = - \frac{Gr}{{\left( {1 + \alpha_{1} q} \right)}}\left[ {\frac{1}{q} - \frac{a}{b + q}} \right] \cdot e^{{ - y\sqrt {\frac{{\Pr (q + \gamma a_{1} )}}{{a_{1} }}} }} . $$

After solving Eq. (16) and introduce the related transform IC and BC, we acquired

17 $$ \begin{gathered} \overline{u}\left( {y,q} \right) = \frac{Gr}{{\left[ {a_{2} + q(a_{6} + a_{5} q)} \right]}}\left( {\frac{1}{q} - \frac{a}{q + b}} \right) \cdot e^{{ - y\sqrt {\frac{q}{{1 + \alpha_{1} q}}} }} - \frac{Gr}{{\left[ {a_{2} + q(a_{6} + a_{5} q)} \right]}}\left( {\frac{1}{q} - \frac{a}{q + b}} \right) \cdot e^{{ - y\sqrt {\frac{{\Pr \left( {\gamma a_{1} + q} \right)}}{{a_{1} }}} }} , \hfill \\ \, \hfill \\ \end{gathered} $$

where $$a_{0} = \frac{1}{{\alpha_{1} }}, \, a_{2} = \Pr \gamma ,a_{3} = \Pr \gamma \alpha_{1} , \, a_{4} = \frac{\Pr }{{a_{1} }}, \, a_{5} = \frac{{\Pr \alpha_{1} }}{{a_{1} }}, \, a_{6} = a_{3} - a_{4} - 1.$$

The inverse LT of Eq. (17) and utilizing the Convolution product, we acquired

18 $$ \begin{aligned} u &=  Gr\left( {\int\limits_{0}^{t} {(t - x)v\Delta \left( {\,y,t,a_{0} ,\alpha_{1} } \right)dx - } \int\limits_{0}^{t} {(t - x)v\Gamma \left( {\,y,\,\,t,\,a_{0} ,\alpha_{1} } \right)dx} } \right) \\ &\quad - Gr\left( {\int\limits_{0}^{t} {v(t - x)\Phi \left( {y,t,a_{1} ,\gamma ,\Pr } \right)dx - } \int\limits_{0}^{t} {v(t - x)\Theta \left( {y,t,a_{1} ,\gamma ,\Pr } \right)dx} } \right), \\ \end{aligned} $$

where

19 $$ \Delta \left( {y,t,a_{0} ,\alpha_{1} } \right) = L^{ - 1} \left\{ {\frac{1}{q} \cdot e^{{ - y\sqrt {\frac{{qa_{0} }}{{\left( {a_{0} + q} \right)}}} }} } \right\} = H(t)\left[ {1 - \frac{{2a_{0} }}{\pi }\int\limits_{0}^{\infty } {\frac{{\sin \left( {yx/\sqrt {\alpha_{1} } } \right)}}{{x\left( {a_{0} + x^{2} } \right)}} \cdot e^{{\left( {\frac{{a_{0} tx^{2} }}{{a_{0} + x^{2} }}} \right)}} dx} } \right], $$

20 $$ v(t) = - \frac{{e^{{t\left( { - \frac{{a_{6} }}{{2a_{5} }} - \frac{{\sqrt { - 4a_{2} a_{5} + a_{6}^{2} } }}{{2a_{5} }}} \right)}} - e^{{t\left( { - \frac{{a_{6} }}{{2a_{5} }} + \frac{{\sqrt { - 4a_{2} a_{5} + a_{6}^{2} } }}{{2a_{5} }}} \right)}} }}{{\sqrt { - 4a_{2} a_{5} + a_{6}^{2} } }}. $$

### Model validation

(i) Considering ${\frac{\partial T\left(y,t\right)}{\partial y}|}_{y=0}=-\left(T\left(0,t\right)+1\right)$ condition of fluid temperature in place of $T(y,t) = 1 - ae^{ - bt} \,$ we get;
  21 $$ \begin{aligned} T(y,t) &=  T_{1} {\text{(y,t)* }}T_{2} {\text{(y,t) 0 < }}\alpha { < 1,}\;{\text{where }} \\ T_{1} {\text{(y,t)}} &=  \delta (t)\int\limits_{0}^{\infty } {e^{{ - ua_{0} \Pr }} \left[ {\frac{1}{{\sqrt {\pi t} }} + e^{u} (2 - erfc(\sqrt u ))} \right]} du \\ &\quad + \int\limits_{0}^{\infty } {\left[ {\frac{1}{{\sqrt {\pi t} }} + e^{u} (2 - erfc(\sqrt u ))} \right]} \sqrt {\frac{{u\alpha a_{0}^{2} \Pr }}{t}} e^{{ - ua_{1} \Pr - \alpha a_{0} t}} I_{0} \left( {2\sqrt {u\alpha a_{0}^{2} \Pr t} } \right)du \\ T_{2} {\text{(y,t)}} & =  1 - \frac{2\Pr \gamma }{\pi }\mathop \smallint \limits_{0}^{\infty } \frac{{{\text{sin}}\left( {yu} \right)}}{{u\left( {\Pr \gamma + u^{2} } \right)}}\exp \left( {\frac{{ - \alpha \gamma tu^{2} }}{{\Pr \gamma + u^{2} }}} \right)du. \, \\ \end{aligned} $$

The result given in (21) is uniform to the published literature given in Ref.^22^.

## Discussion and results

**Figure 2:**
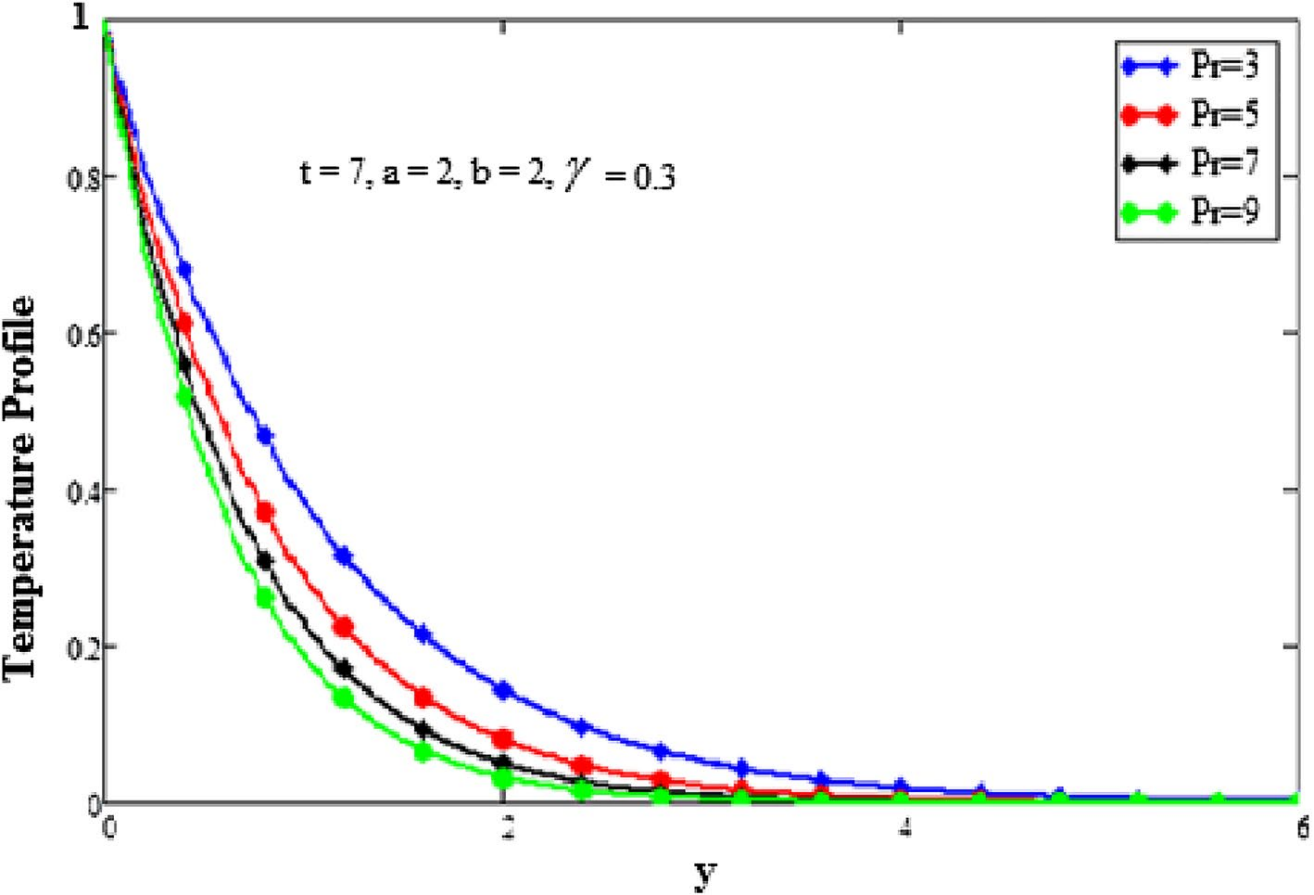
Analysis of Prandtl numer Pr on temperature profile.

**Figure 3:**
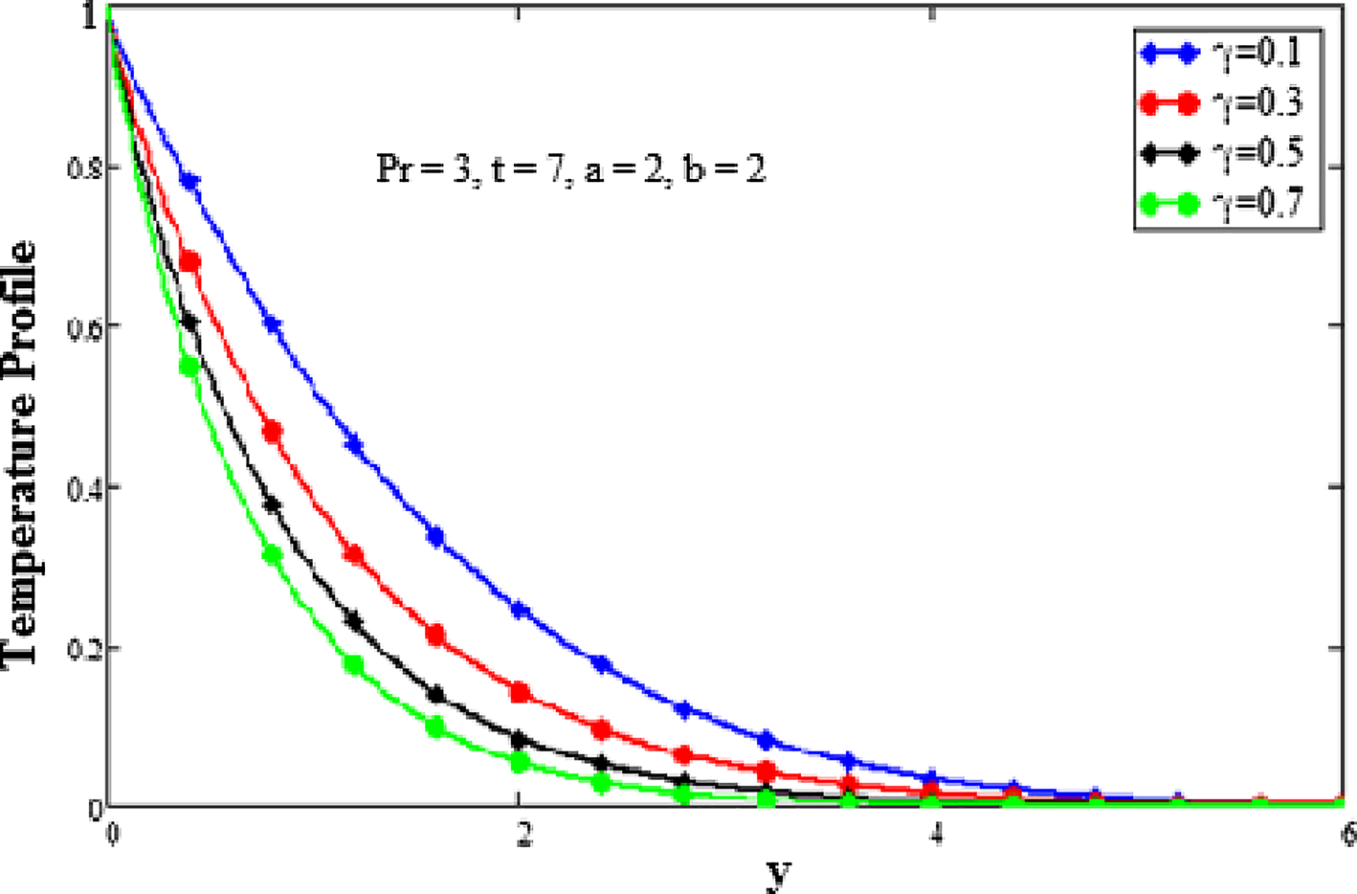
Analysis of fractional parameter $\gamma$ on temperature profile.

**Figure 4:**
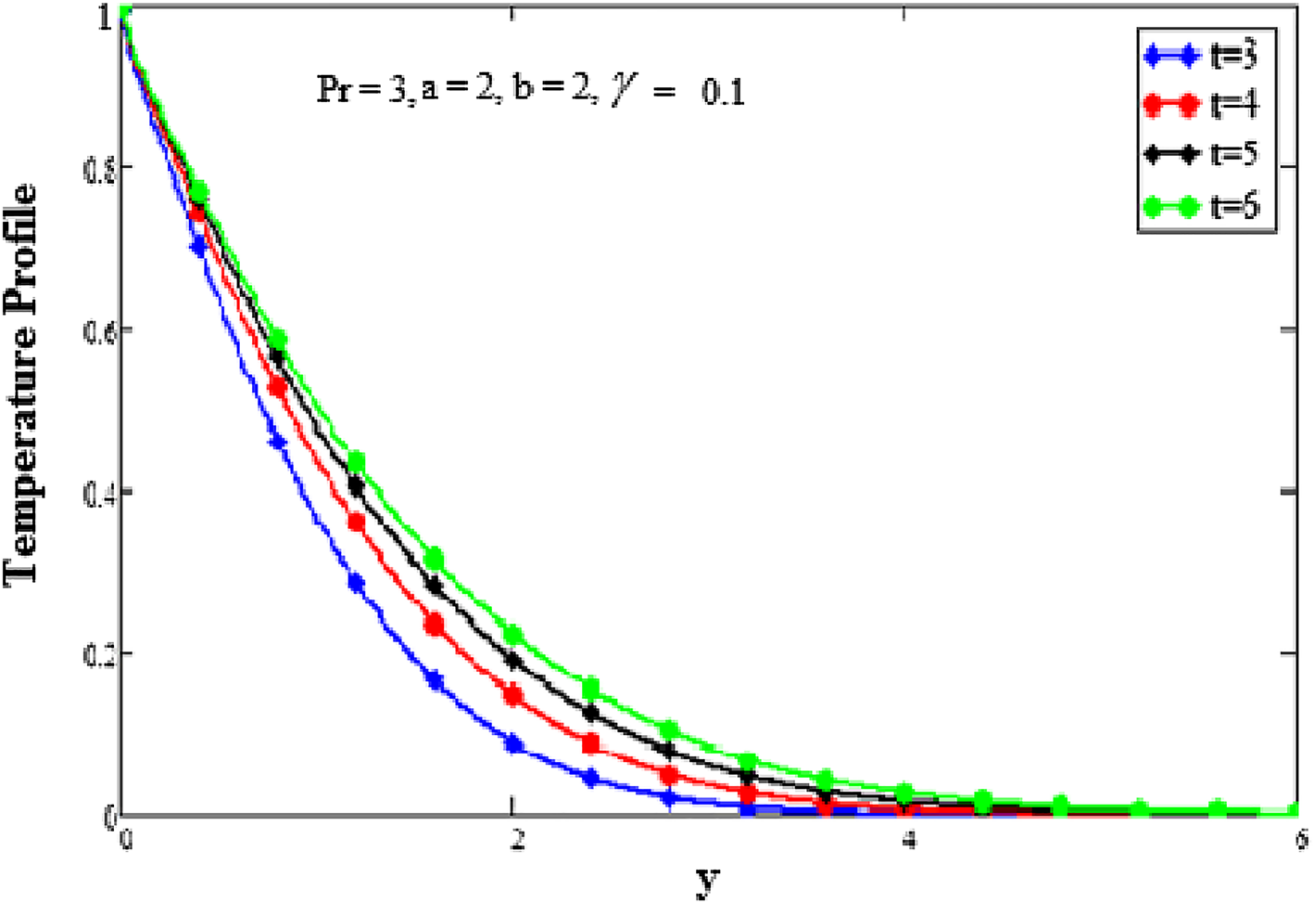
Analysis of time t on temperature profile.

In this section, we examine physical comprehension. The physical sketch of the problem is given in Fig. 1. To determine the effects of various embedded components, such as the Grashof number, parameter for second-grade fluid, the Prandtl number, the fractional parameter, and the time, certain mathematical calculations have been made. Every graph is plotted against y. The effects of different factors on the temperature profile are shown in Figs. 2, 3 and 4. The behavior of the Prandtl number on the temperature field is shown in Fig. 2. It shows that when the Prandtl number increases, the temperature profile slows down. Physically, this phenomenon results from a drop in thermal conductivity as a result of a drop in fluid temperature. The dimensionless Prandtl number is an intrinsic characteristic of the fluid. The free flowing fluids have small Prandtl number with high heat conductivity. The Prandtl number controls the relative thickness of the thermal and momentum boundary layers. The Prandtl number is the ratio of the momentum diffusivity to the thermal conductivity so when the Prandtl number increases it causes a decrease in the thermal conductivity. Hence the temperature of the fluid decreases with the increase in the Prandtl number. The effects of a fractional parameter on temperature are plotted in Fig. 3. When the values of the fractional parameter increases the thermal boundary layer thickness increases. Therefore when the values of fractional parameter increases, the fluid's temperature decreases.

**Figure 5:**
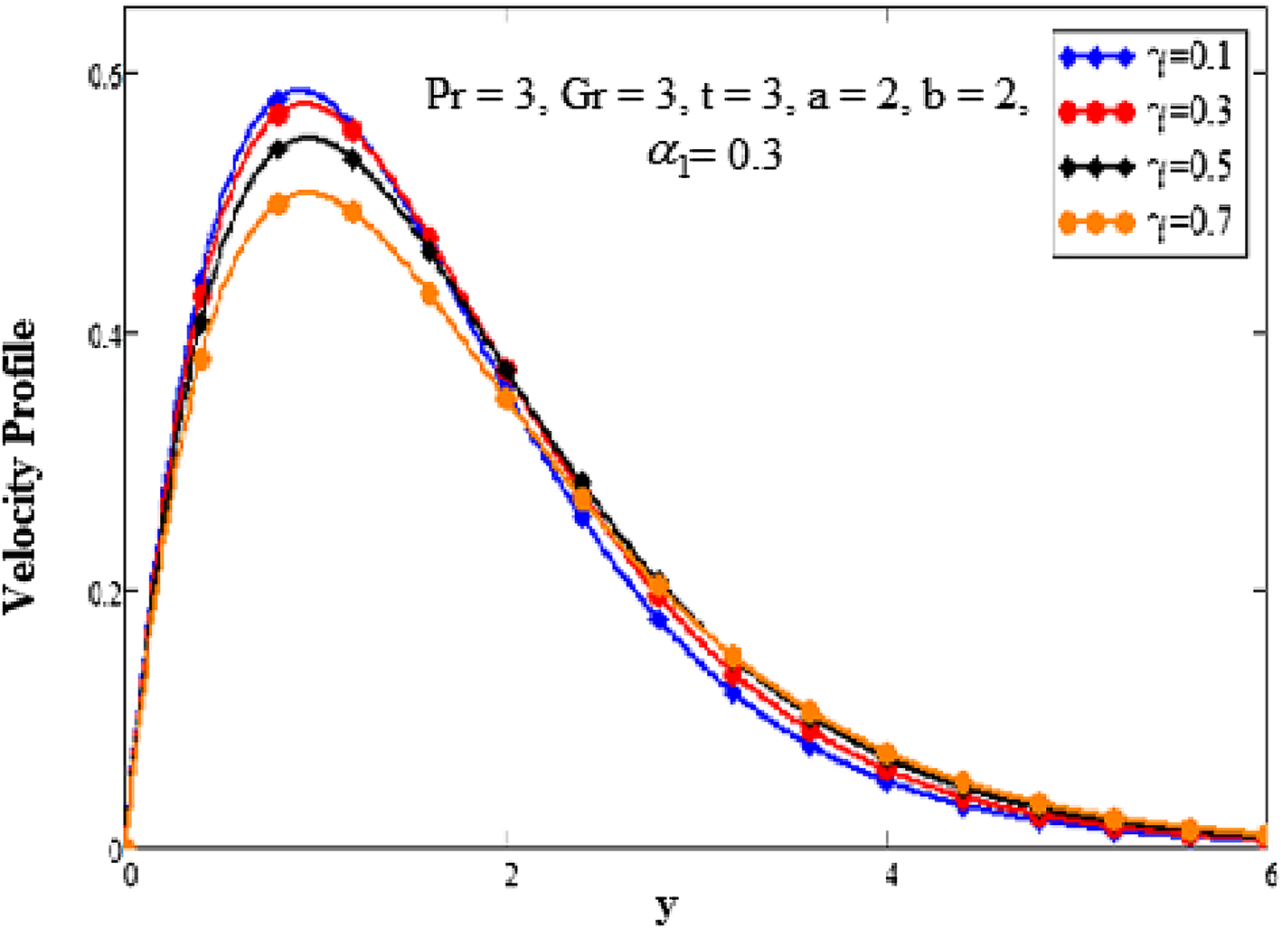
Analysis of fractional parameter $\gamma$ on velocity profile.

**Figure 6:**
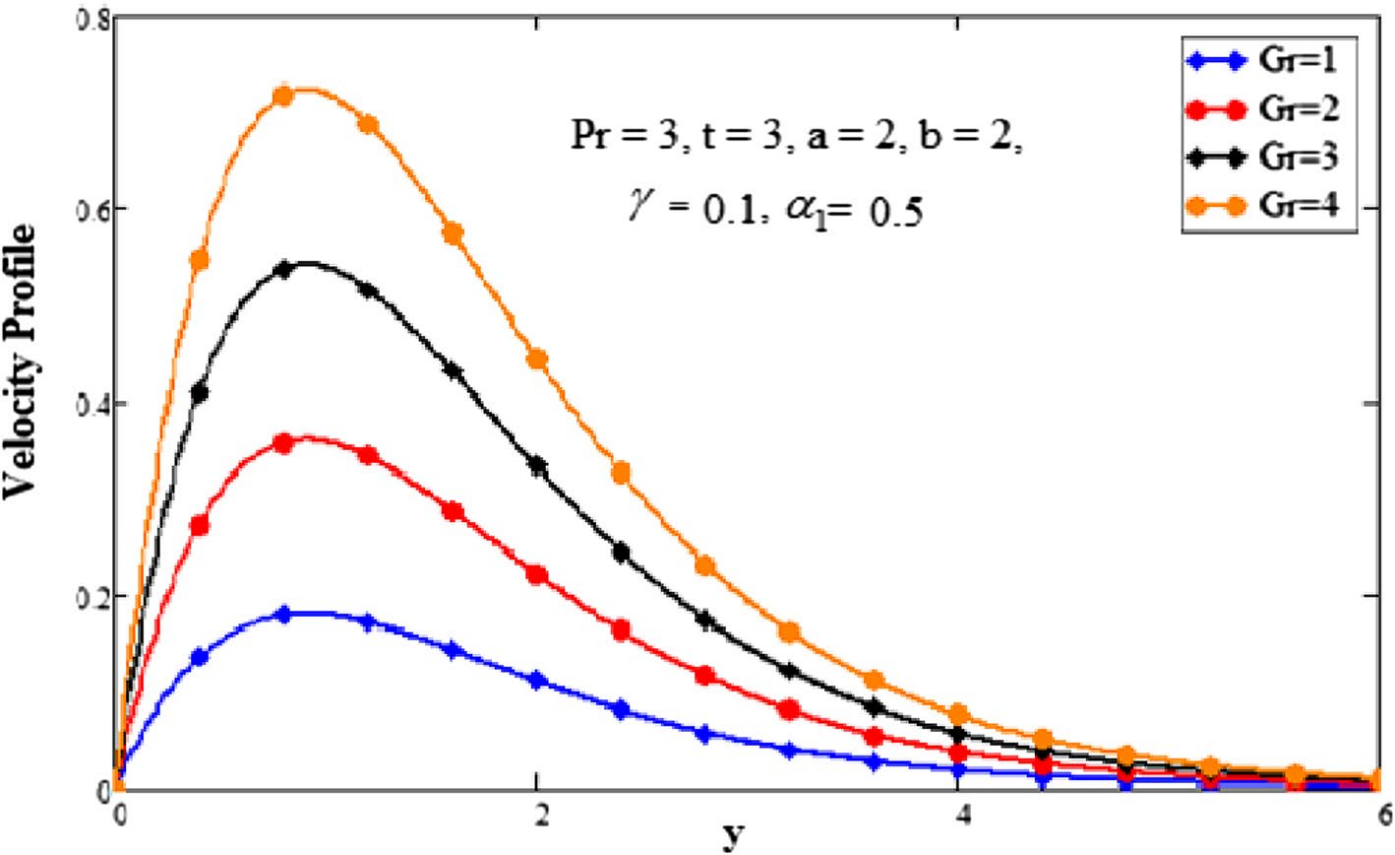
Analysis of Grashof number Gr on velocity profile.

**Figure 7:**
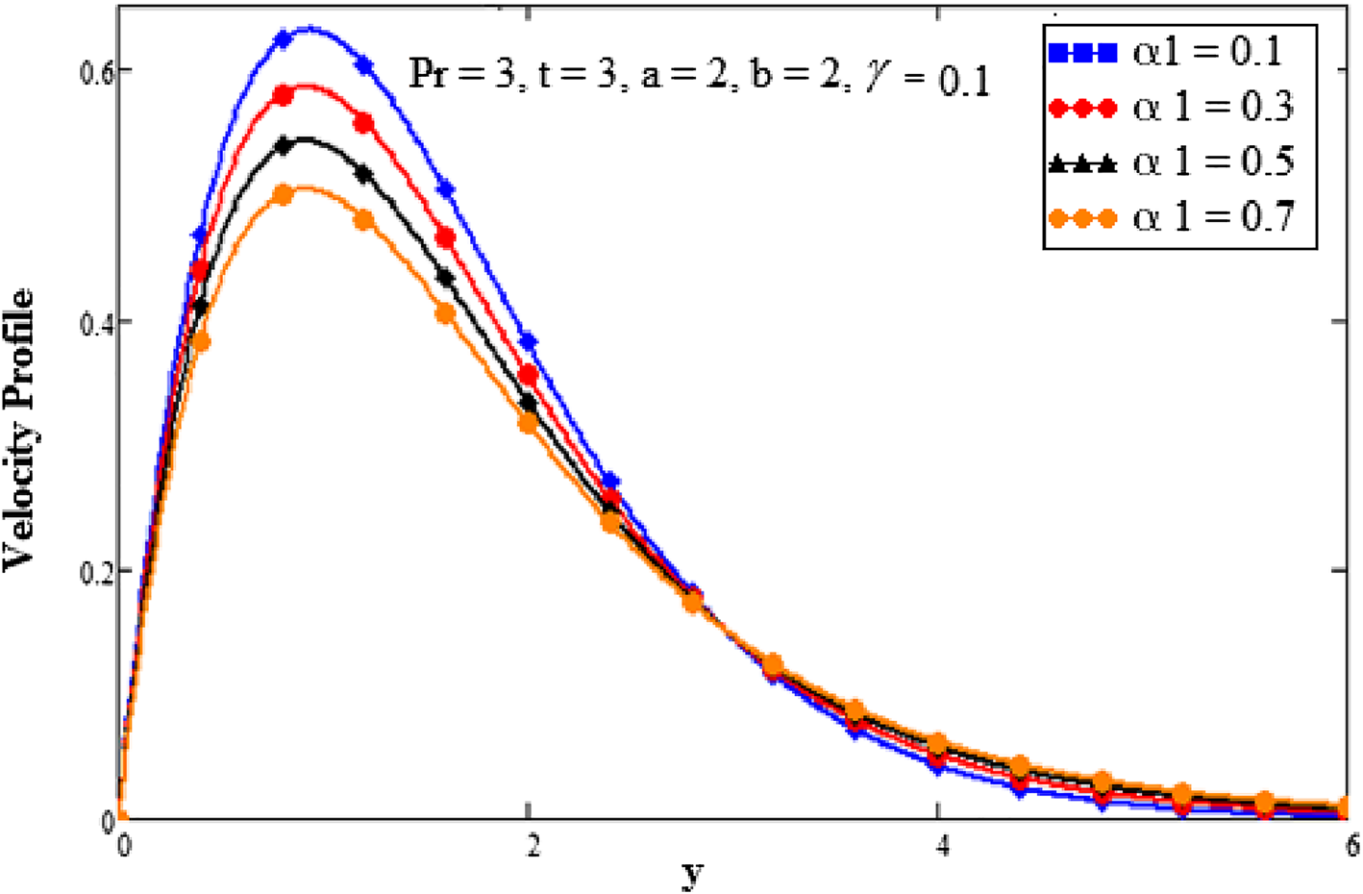
Analysis of second grade parameter $\alpha_{1}$ on velocity profile.

**Figure 8:**
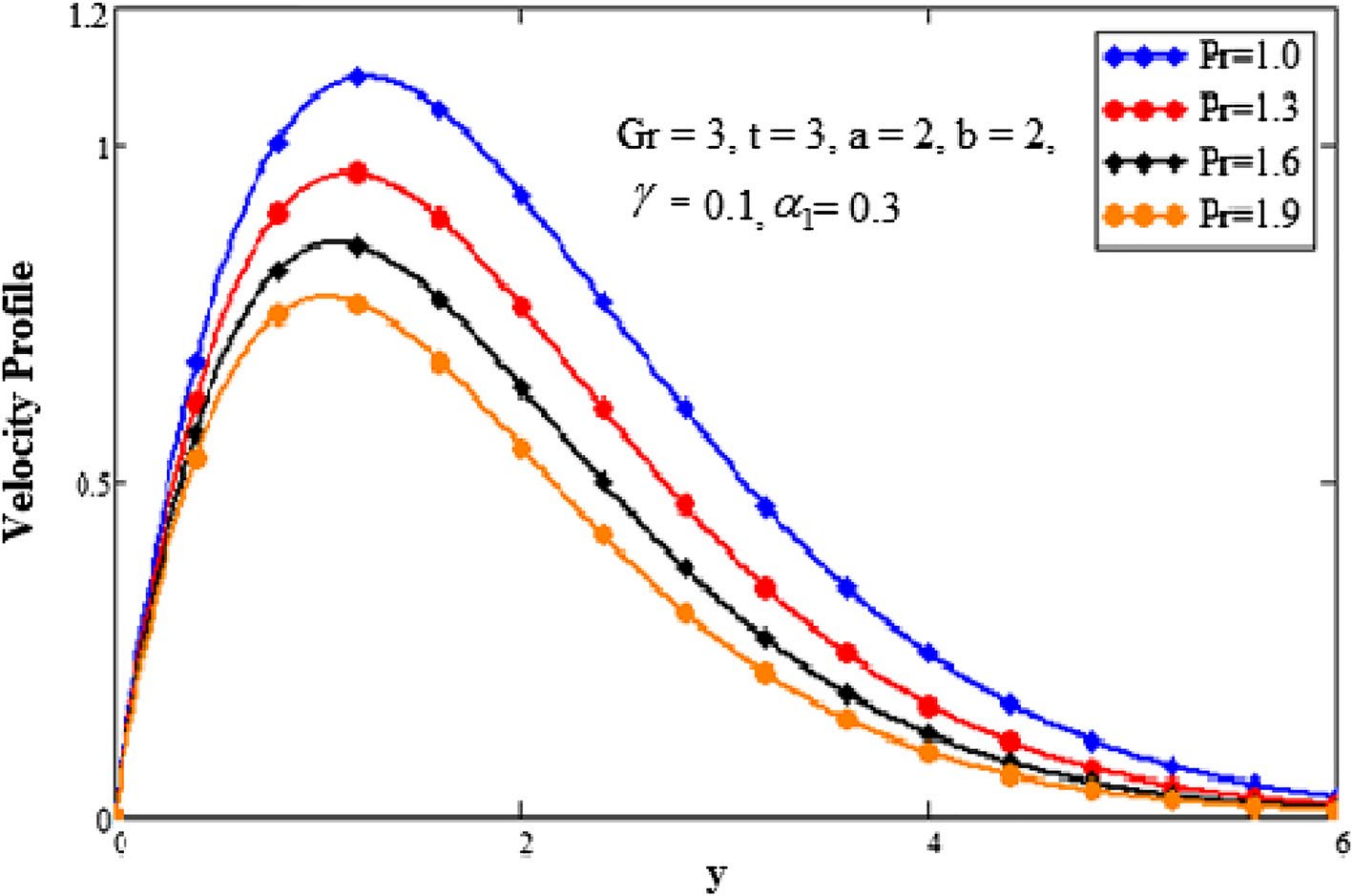
Analysis of Prandtl numer Pr on velocity profile.

**Figure 9:**
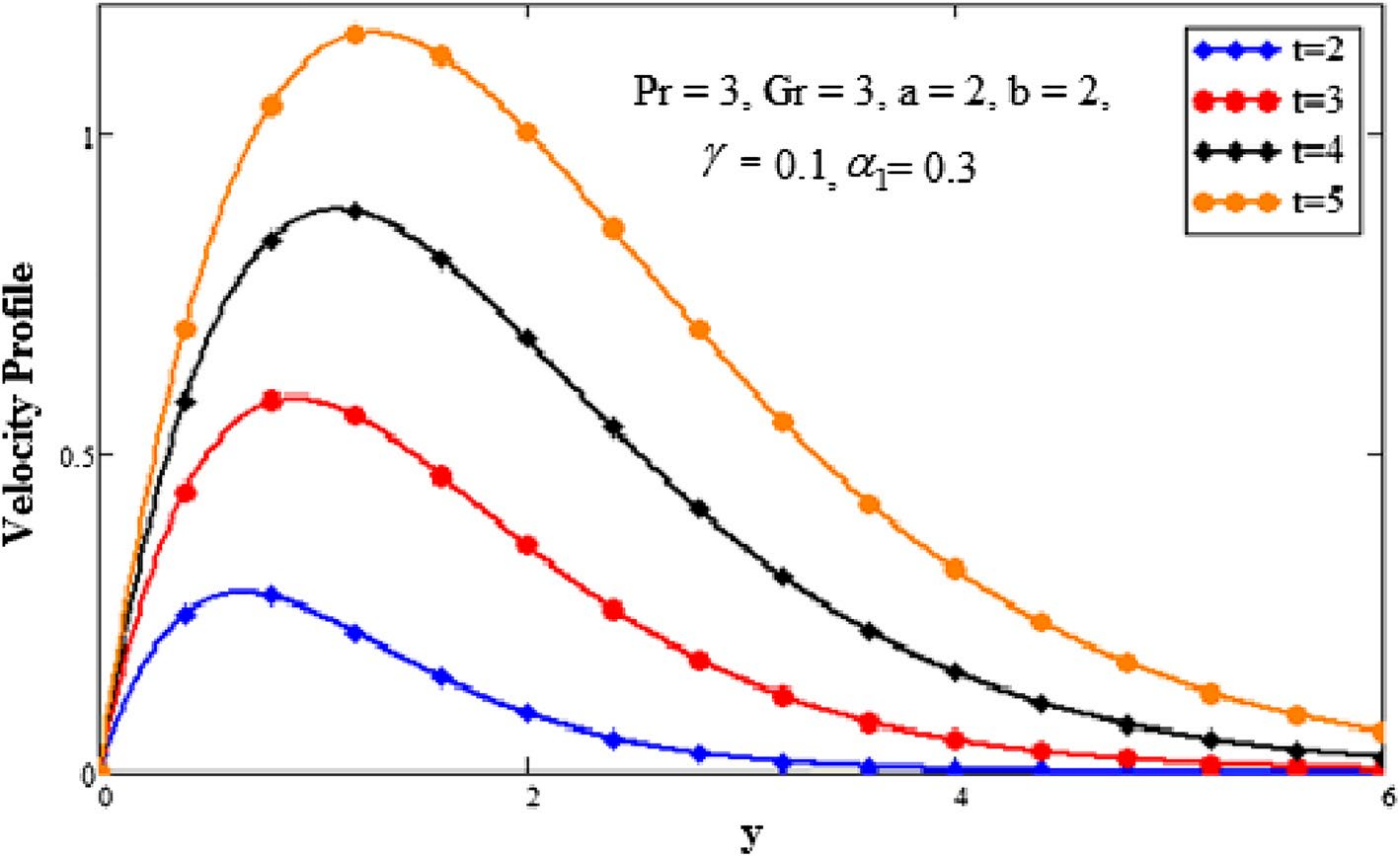
Analysis of time t on velocity profile.

The effects of time on the temperature profile are depicted in Fig. 4 plots. It has been noted that as the value of time increases, so does the temperature profile. We have discovered that as the values of time are increased, the boundary layer becomes thicker. Close to the plate, when it asymptotically approaches zero, is where the temperature is the highest. The behavior of several embedded elements on velocity profiles is depicted in Figs. 5, 6, 7, 8 and 9. The impact of a fractional parameter on the velocity field is depicted in Fig. 5. When the values of the fractional parameter increases the momentum boundary layer thickness increases. So, with increasing values of the fractional factor, it can be seen that the fluid's velocity decreases. After a certain threshold for y, they exhibit the opposite effects, i.e., the velocity increases as the value of the fractional parameter is increased.

Figure 6 depicts the properties of the Grashof number. For larger values of the Grashof number, the fluid’s velocity increases. Since the Grashof number is the ratio of the buoyancy forces to the viscous forces so the increasing values of the Grashof number, decreases the viscosity and as consequence of decrease in viscosity the velocity of the fluid increases. The Grashof number is used to describe the association of the mass and heat transfer thermally induced by natural convection. Physically, this is due to the forces of viscosity and thermal upgrading. The existence of convective current electrified the fluid more as the Grashof number increased. Convective current boosts the fluid’s velocity via increasing buoyancy force.

The effect of the second-grade fluid component on velocity is seen in Fig. 7. With a higher value of second-grade fluid, the fluid’s motion decreases. This demonstrates the fall in momentum limit layer thickness. The effect of the Prandtl number on the momentum limit layer is seen in Fig. 8. It shows that the fluid velocity decreases as the Prandtl number rises. The Prandtl number is the ratio of the momentum diffusivity to the thermal conductivity so when the Prandtl number increases it causes an increase in the momentum diffusivity. The Prandtl number and heat conductivity have an inverse relationship. The fluid will have lower heat conductivity for larger Prandtl numbers, which results in a higher viscosity. The velocity decreases as a result. Figure 9 emphasizes how time affects the velocity field. It has been noted that when time goes faster, fluid velocity also accelerates. Fluid velocity increases closer to the plate and as we move away from it, it asymptotically decreases to zero.

## Conclusions

We looked into the generalized Fourier law, thermal heat flux, and natural convection flow of a second-grade fluid in this paper’s for closed-form solution. Using the Caputo–Fabrizio fractional derivative, the well-known model is converted to a fractional model. The Laplace transform is used to obtain the solutions to the momentum and energy equation. Plots are made for a number of embedded parameters that are part of the momentum and energy equation. The following list includes the thesis’ principal conclusions:

- The temperature of the fluid decreases with the increasing values of the Prandtl number and fractional parameter.
- The temperature of the fluid increases with the increasing values of the time.
- The velocity of the fluid decreases with the increasing values of the fractional parameter.
- The velocity of the fluid increases with the increasing values of the Grashof number and time.
- The velocity of the fluid decreases with the increasing values of the second grade parameter and Prandtl number.

### Future recommendation

- This problem can be extended by solving this problem for Heat transfer analysis of generalized second-grade fluid with exponential heating and non-Fourier heat flux.
- This problem can be extended by solving this problem by adding heat generation and radiation source to the same problem.
- This problem can be extended by solving this problem by adding concentration class to the same problem.

## Data Availability

The datasets analyzed during the current study are available from the corresponding author on reasonable request.
